# Case Report: Urethral Diverticulum after Distal Hypospadias Repair: An Uncommon Complication.

**DOI:** 10.12688/f1000research.148191.1

**Published:** 2024-03-27

**Authors:** Ammar Kheyami, Abdulla AlMuharraqi, Mahmood Abbas, A.K Singhal

**Affiliations:** 1 Pediatric Surgery, Ibn Alnafees Hospital, Manama, Bahrain; 2 General Practice, Manama Medical Center, Manama, Bahrain

**Keywords:** Urethra, diverticulum, hypospadias, circumcision.

## Abstract

**Introduction:**

A urethral diverticulum can be defined as a pocket that forms from the lining of the urethra and protrudes into the surrounding tissue, a condition which causes voiding dysfunction and may result as a rare complication of hypospadias repair surgery.

**Case report:**

We report the case of a 2-year-old child who presented to us in 2019 complaining of a thin forceful stream, ballooning of the ventral aspect of the penis while voiding, and post-void dribbling. He has a history of undergoing a tabularised incised plate urethroplasty for distal penile hypospadias at 18-months-old. Ultrasound showed increased post-void residual volume and cystourethroscopy confirmed a urethral diverticulum extending from the subcorona to the base of the penis. The patient underwent partial excision of diverticulum, urethroplasty, and meatoplasty. He was followed-up 3 months later with complete resolution of his symptoms and a normal urinary stream with no urethral ballooning or dribbling.

**Conclusion:**

Urethral diverticulum may present as a complication post hypospadias repair. Although it is rare, we believe that it is important for the patient’s parents to understand the possibility and know of the signs and symptoms in addition to attending regular outpatient clinic appointments in order to facilitate early management if needed. Furthermore, it is highly important for physicians to assess newborns for hypospadias before carrying out circumcision as it is a contraindication for the procedure.

## Introduction

Urethral diverticulum - an out-pouching of the urethra into surrounding tissues - is an important but uncommon complication of hypospadias repair. ^1^ Studies suggest that it comprises 0.3% of postoperative complications and is mainly associated with proximal hypospadias. ^2^^,^^3^ Factors predisposing to its formation include proximal defect, oversized neourethra, poorly supporting tissue covering urethroplasty, and distal urethral stricture. ^2^^,^^3^ We present a case of postoperative urethral diverticulum formation after distal hypospadias repair and discuss possible treatment options.

## Case report

**Figure 1.**
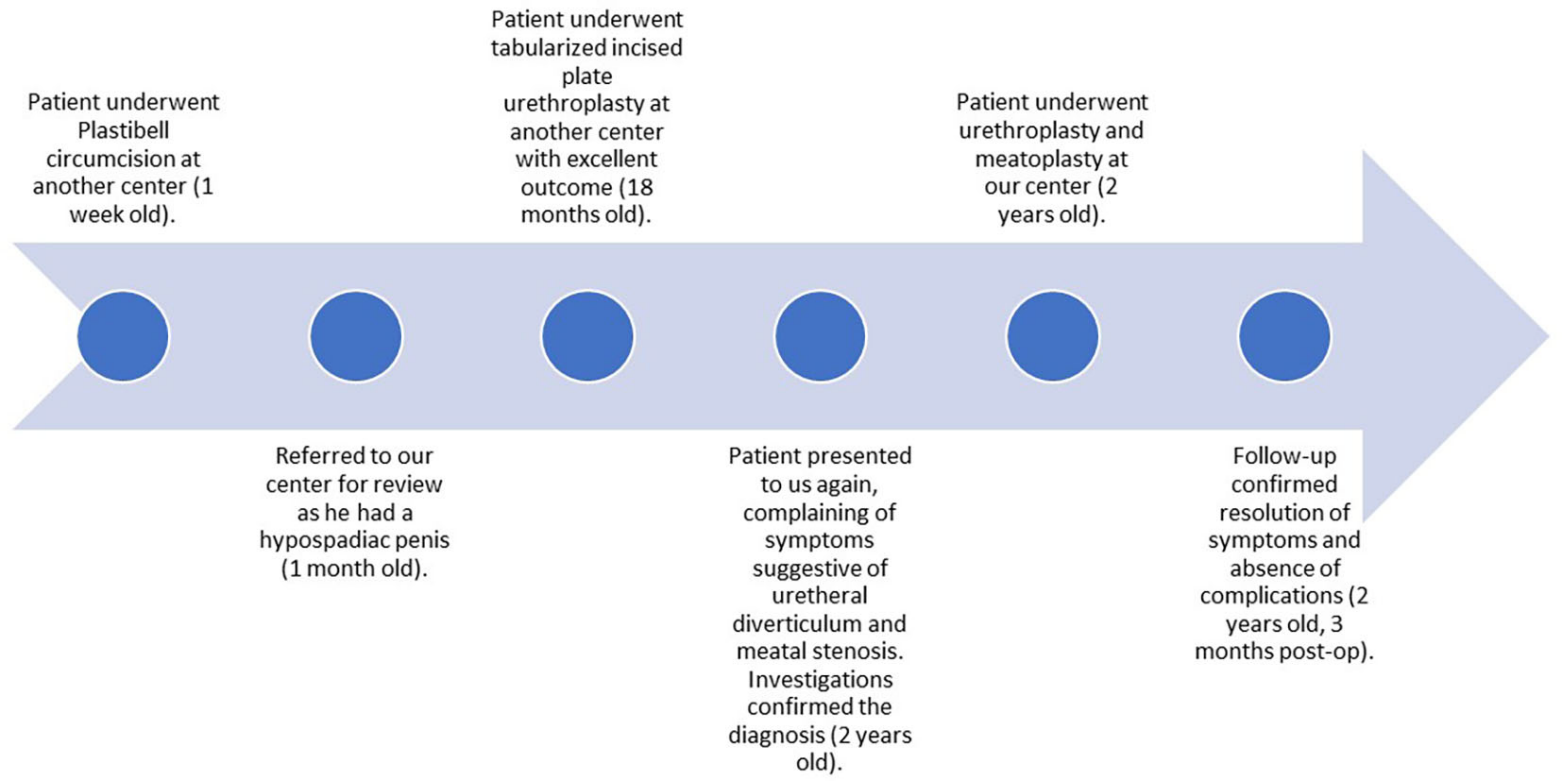
A timeline of the patient’s presentations and the procedures he underwent.

**Figure 2.**
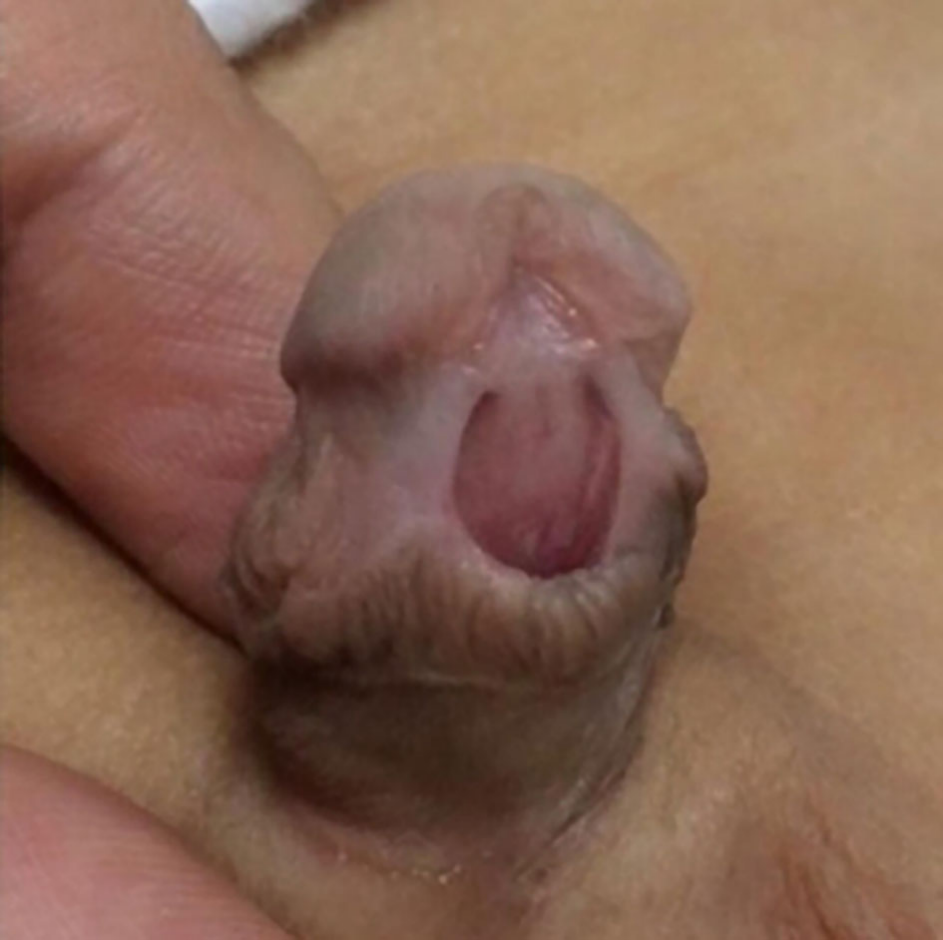
Ventral view of the penis (excessively circumcised with a distal penile patulous meatus).

The child was one-month-old ( Figure 1) when referred to our centre in 2019 for review after undergoing Plastibell circumcision. On examination, he was circumcised with a hypospadiac meatus located at distal penile position ( Figure 2).

**Figure 3.**
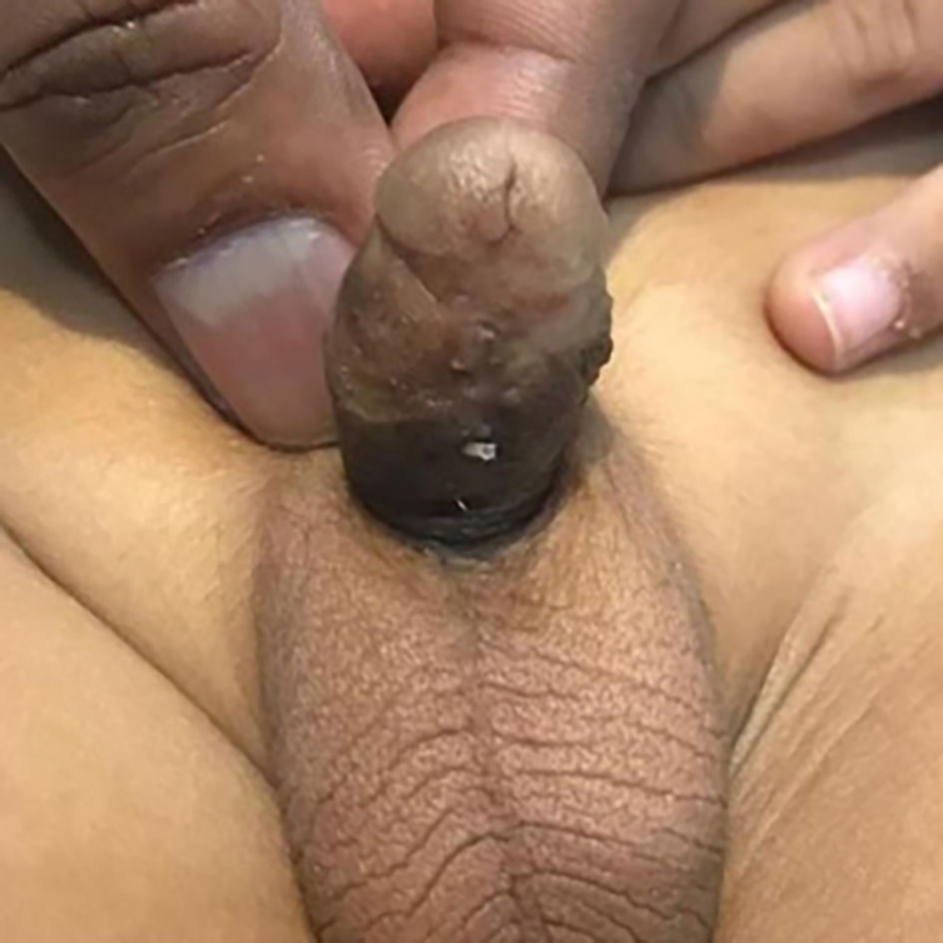
Ventral view of the penis after tabularised incised plate urethroplasty.

At 18 months of age, he underwent a tabularised incised plate urethroplasty at an external paediatric urology centre with an excellent outcome ( Figure 3).

**Figure 4.**
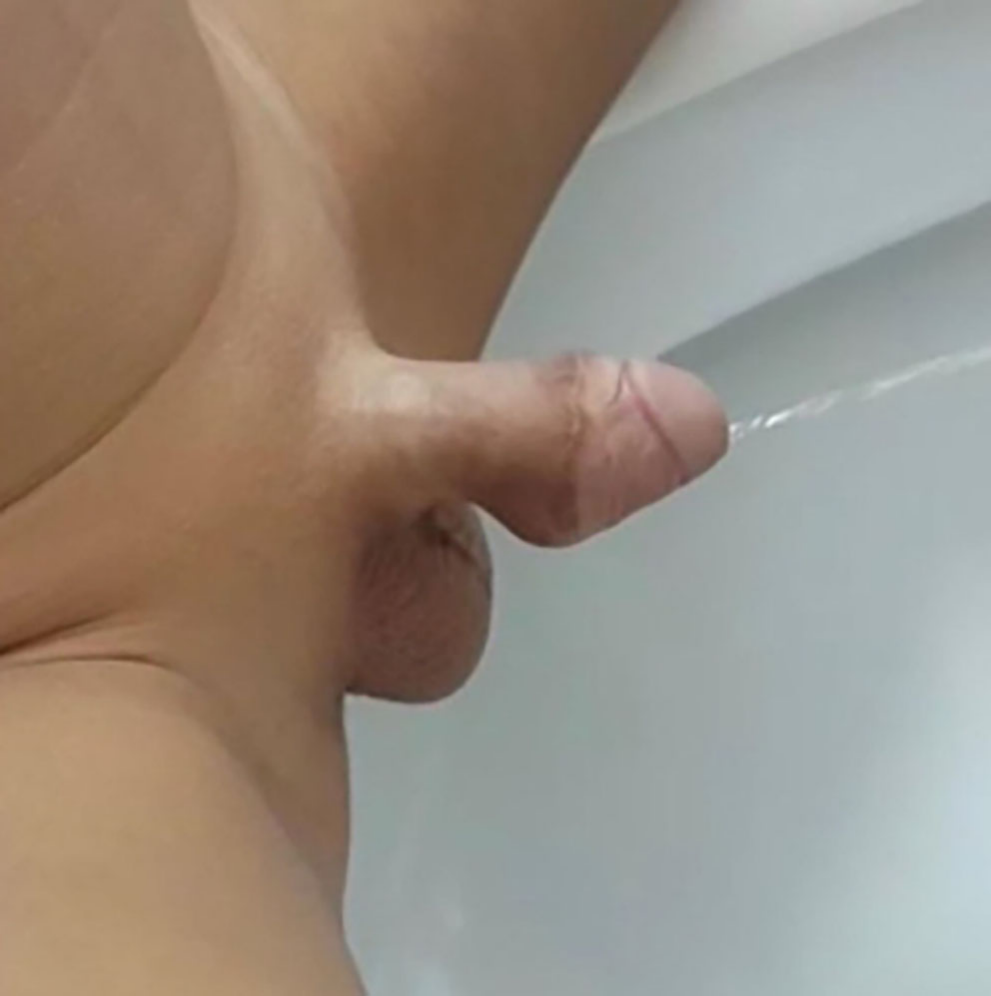
Penis with urethral diverticulum.

One year later, he presented with typical signs and symptoms of a urethral diverticulum and meatal stenosis; thin forceful stream, ballooning of the ventral aspect of the penis during voiding, significant post-void dribbling, and need for manual emptying of the diverticulum ( Figure 4).

**Figure 5.**
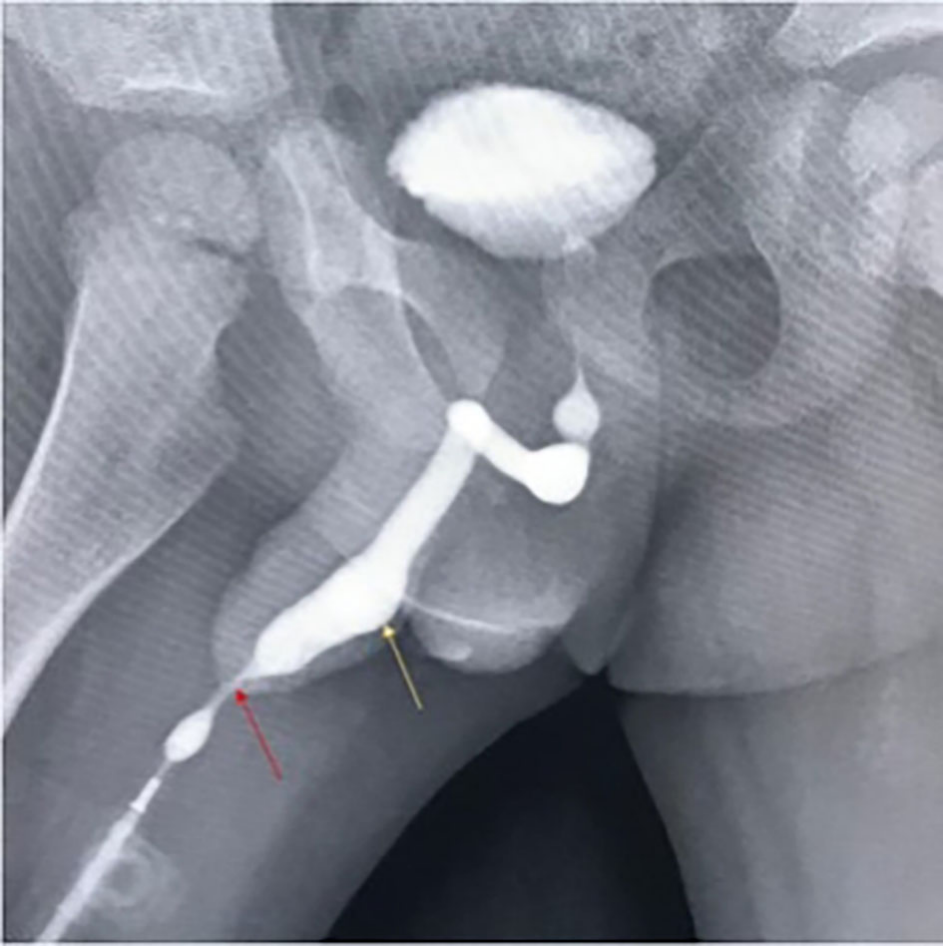
Ascending urethrogram showing meatal narrowing (red arrow) and a proximal urethral diverticulum (yellow arrow).

**Figure 6.**
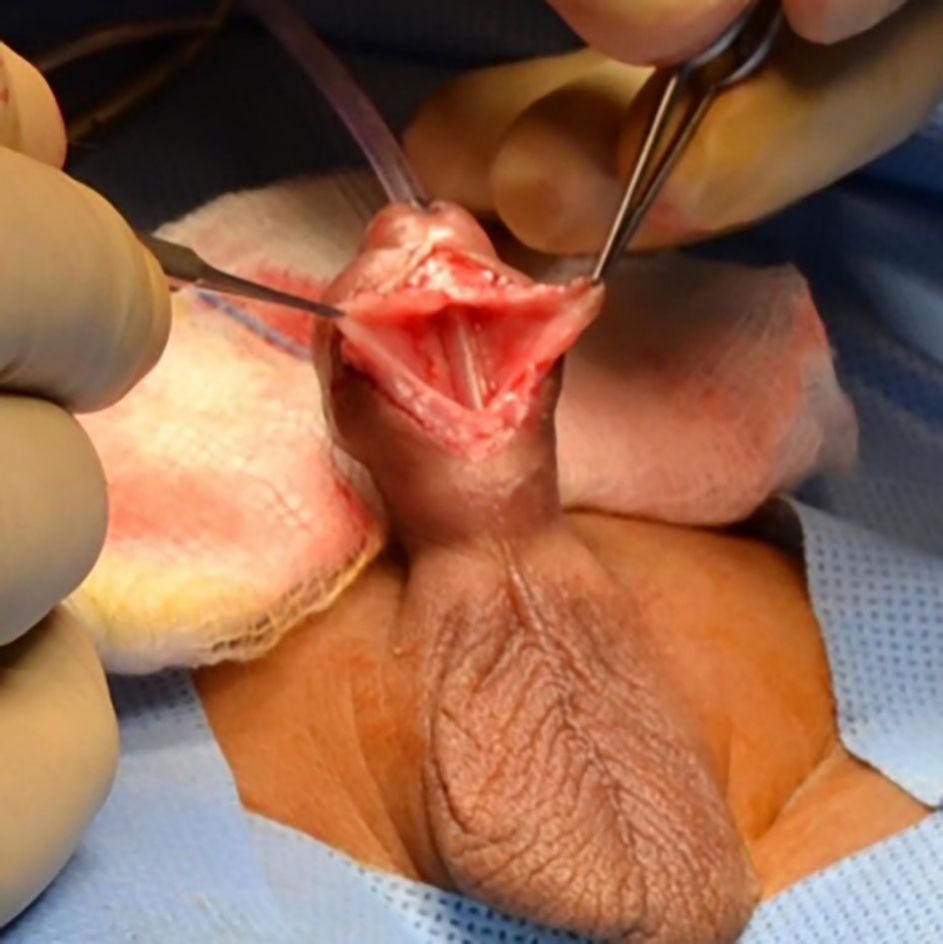
Ventral view of the penis during standard meatoplasty. The meatus was opened wider and stitches were taken at the margins.

Ultrasound showed diffuse bladder wall thickening with significant post-void residual volume. Ascending urethrogram confirmed the diagnosis of meatal stenosis with proximal diverticulum ( Figure 5). Patient underwent cystourethroscopy which confirmed a diverticulum extending from the subcorona to the base of the penis. The diverticulum was opened through a ventral midline incision. A midline strip of 16 mm width of mucosa was preserved, and lateral mucosa was excised preserving the underlying dartos tissue ( Figure 6).

The urethroplasty was closed in two layers and was supported by double-breasted Dartos fascia. At the end of the procedure, standard meatoplasty was performed for meatal stenosis. Three months following repair, the child had a normal thick continuous stream with no post-void dribbling or ballooning of the urethra.

## Discussion

Urethral diverticulum is an uncommon complication of hypospadias surgery which has several risk factors that could influence its incidence such as a large neourethra, inadequate supporting tissue post-urethroplasty, and a distal urethral stricture. ^2^^,^^3^ It can be diagnosed via clinical evaluation and confirmed with cystourethrography and cystoscopy. Partial excision of diverticulum and urethroplasty is an acceptable form of treatment for such cases, as the final aim is to restore a urethra with normal diameter and pressure that is also well supported with enough tissue and no distal obstruction. ^2^^,^^4^ In the presented case, the diagnosis of hypospadias was missed at birth and a circumcision was carried out though circumcision is contraindicated in these cases. This certainly requires more awareness since the repair of hypospadias in uncircumcised children is technically easier and has a better outcome. Additionally in this case, excessive circumcision resulted in a very wide meatus along with a severe deficiency in the ventral skin and the underlying supportive tissue which possibly complicated the repair and played an important role in the formation of the diverticulum. Meatal stenosis is another factor that predisposed to diverticulum formation in this child, and it was most likely a post-surgical complication as it can occur after any hypospadias surgery. Therefore, regular follow-up after hypospadias repair is of vital importance for early diagnosis and management of meatal stenosis.

## Conclusion

Meatal stenosis and urethral diverticulum are amongst the possible complications of hypospadias repair. The repair of urethral diverticula is challenging, and the outcome depends on the creation of a well-supported and normal-sized urethra with no distal obstruction. Furthermore, parents should be educated about the signs and symptoms of meatal stenosis and the need for strict and regular follow-up to diagnose and manage any complication as early as possible. It is important to carefully examine the child for hypospadias before circumcision as it is contraindicated.

## Consent

We discussed and explained to the patient’s father about the writing and publication of this case report. He had the opportunity to ask questions about everything related to this process, and understood that no identifying information related to the patient will be shared; Written informed consent for the publication of patient details and images was obtained from the patient’s father.

## Data Availability

All data underlying the results are available as part of the article and no additional source data are required.
