# Supplementary material for: Case Report: Urethral Diverticulum after Tubularised Incised Plate Repair of Distal Hypospadias: An Uncommon Complication
Source: F1000Res. 2024 Jun 28;13:222. Originally published 2024 Mar 27. [Version 2] doi: 10.12688/f1000research.148191.2 (PMC11231625; doi:10.12688/f1000research.148191.2)
Supplement: Peer review history

# Reviewer report: Reviewer response for version 2

**DOI:** 10.5256/f1000research.168132.r296925

**Published:** 2024-07-08

**Authors:** Mohamed Zouari

**Affiliations:** 1 University of Sfax, Sfax, Tunisia

The authors reported a case of postoperative urethral diverticulum following distal hypospadias repair in a child and discussed treatment options of this rare condition.

 The authors have responded to all comments and made the required corrections. I recommend accepting the manuscript for indexing in its present form.

Are enough details provided of any physical examination and diagnostic tests, treatment given and outcomes?

Yes

Is the case presented with sufficient detail to be useful for other practitioners?

Yes

Is sufficient discussion included of the importance of the findings and their relevance to future understanding of disease processes, diagnosis or treatment?

Partly

Is the background of the case’s history and progression described in sufficient detail?

Yes

Reviewer Expertise:

Pediatric surgery

I confirm that I have read this submission and believe that I have an appropriate level of expertise to confirm that it is of an acceptable scientific standard.

# Reviewer report: Reviewer response for version 2

**DOI:** 10.5256/f1000research.168132.r296926

**Published:** 2024-07-04

**Authors:** Johannes Wirmer

**Affiliations:** 1 Sana Klinikum, Offenbach, Germany

I have no further suggestions

Are enough details provided of any physical examination and diagnostic tests, treatment given and outcomes?

Yes

Is the case presented with sufficient detail to be useful for other practitioners?

Yes

Is sufficient discussion included of the importance of the findings and their relevance to future understanding of disease processes, diagnosis or treatment?

Partly

Is the background of the case’s history and progression described in sufficient detail?

Yes

Reviewer Expertise:

Hypospadiology

I confirm that I have read this submission and believe that I have an appropriate level of expertise to confirm that it is of an acceptable scientific standard.

# Reviewer report: Reviewer response for version 1

**DOI:** 10.5256/f1000research.162472.r261657

**Published:** 2024-04-30

**Authors:** Mohamed Zouari

**Affiliations:** 1 University of Sfax, Sfax, Tunisia

Urethral diverticula can be congenital or acquired; the most common cause of an acquired diverticulum in children is previous hypospadias repair. The reported incidence of diverticula formation after hypospadias repair is 0–21% [Radojicic ZI, et. al., 2004 (Ref 1)]. Factors increasing the risk of diverticula include a proximal defect, use of poorly supported tissue for interposition grafting (e.g. bladder mucosa or tubularized pedicle grafts), and the presence of a distal stricture resulting in relative obstruction/higher outlet pressures [Horton CE, et. al., 1998 (Ref 2), Snyder CL, et. al., 2005 (Ref 3)].

 In this article, the authors reported the case of a urethral diverticulum in a 2-year-old child circumcised at the neonatal age. This diverticulum occurred after of distal hypospadias repair as a complication of the TIP-technique. Although the article is interesting and the illustrations attractive, I believe that there are two major problems that need to be corrected.

 The first problem concerns Figure 1, which clearly shows a mega-meatus with an intact penis. The authors need to clarify whether or not this was an accidental circumcision, and on what grounds this assumption was made. There are no signs of scarring or trauma to the penis. The penile folds are well-defined, showing that the penis has not been damaged.

 My second problem concerns the English language, which needs revision. For example, I can identify the word "TUBULARIZED" which is written in the article "TABULARISED" several times. Other sentences use non-technical words such as "ballooning of the ventral aspect of the penis".

 In conclusion, this article is interesting since it highlights an uncommon complication of TIP-uretroplasty in children. This complication could lead to therapeutic challenges, for which there is still no consensus on management.

Are enough details provided of any physical examination and diagnostic tests, treatment given and outcomes?

Yes

Is the case presented with sufficient detail to be useful for other practitioners?

Yes

Is sufficient discussion included of the importance of the findings and their relevance to future understanding of disease processes, diagnosis or treatment?

Partly

Is the background of the case’s history and progression described in sufficient detail?

Yes

Reviewer Expertise:

Pediatric surgery

I confirm that I have read this submission and believe that I have an appropriate level of expertise to confirm that it is of an acceptable scientific standard, however I have significant reservations, as outlined above.

## References

[1] : 'Pseudospongioplasty' in the repair of a urethral diverticulum. *BJU Int* .2004;94(1) : 10.1111/j.1464-410X.2003.04913.x 126-30 10.1111/j.1464-410X.2003.04913.x 15217446

[2] : Complications of hypospadias surgery. *Clin Plast Surg* .1988;15(3) :371-9 3134152

[3] : Management of urethral diverticulum complicating hypospadias repair. *J Pediatr Urol* .2005;1(2) : 10.1016/j.jpurol.2004.10.001 81-3 10.1016/j.jpurol.2004.10.001 18947541

# Response

**Published:** 2024-06-19

**Authors:** Abdulla AlMuharraqi

**Affiliations:** General Practice, Manama Medical Center, Manama, Bahrain

Dear Dr. Mohamed,

 Thank you for your review and invaluable comments. Spelling corrections have been made as per your advice.

 In regards to your comment concerning Figure 1, when we discussed the case with the General Practitioner who performed the circumcision, they explained that in their opinion it was a mild case of hypospadias and that the foreskin was intact. However, after the procedure the patient presented with the findings described in the manuscript.

 Kind regards,

 Dr. Abdulla Al-Muharraqi

# Reviewer report: Reviewer response for version 1

**DOI:** 10.5256/f1000research.162472.r261654

**Published:** 2024-04-05

**Authors:** Johannes Wirmer

**Affiliations:** 1 Sana Klinikum, Offenbach, Germany

The Report describes a case of urethral diverticulum after tubularized-inzised plate repair (TIP) of a distal Hypospadias. The Hypospadias repair was performed at the age of 18 month, but had a circumcision as a newborn. From the picture taken before the hypospadias repair, he most likely had a MIP-Hypospadias (megameatus intact prepuce).

 The here described developement of an urethral diverticulum after repair of distal hypospadias is an important complication of the TIP-technique. Even though it has been described before ( Lee H, et al., 2006) [Ref 1], the common assumption is still that it mainly occurs after flap urethroplasty. This article is an important contribution to drawing attention to the complications of tip correction.

 Small suggestions for corrections would be:

 Title: Case Report: Urethral Diverticulum after TIP-Repair of distal hypospadias: a not so uncommon complication

 In addition, tabularized incised plate urethroplasty is mentioned several times throughout the text, where it should correctly be called TUBULARIZED plate urethroplasty.

Are enough details provided of any physical examination and diagnostic tests, treatment given and outcomes?

Yes

Is the case presented with sufficient detail to be useful for other practitioners?

Yes

Is sufficient discussion included of the importance of the findings and their relevance to future understanding of disease processes, diagnosis or treatment?

Partly

Is the background of the case’s history and progression described in sufficient detail?

Yes

Reviewer Expertise:

Hypospadiology

I confirm that I have read this submission and believe that I have an appropriate level of expertise to confirm that it is of an acceptable scientific standard, however I have significant reservations, as outlined above.

## References

[1] : Surgical Correction of Post-hypospadias Repair Urethral Diverticulum and Stricture using Rotational Flap. *Korean Journal of Urology* .2006;47(11) : 10.4111/kju.2006.47.11.1255 10.4111/kju.2006.47.11.1255

# Response

**Published:** 2024-06-19

**Authors:** Abdulla AlMuharraqi

**Affiliations:** General Practice, Manama Medical Center, Manama, Bahrain

Dear Dr. Johannes,

 Thank you for your review and invaluable comments. Changes have been made as per your suggestions.

 Kind regards,

 Dr. Abdulla Al-Muharraqi
